# Prevalence of Antibiotic-Resistant *E. coli* Strains in a Local Farm and Packing Facilities of Honeydew Melon in Hermosillo, Sonora, Mexico

**DOI:** 10.3390/antibiotics11121789

**Published:** 2022-12-09

**Authors:** Yessica Enciso-Martínez, Edwin Barrios-Villa, César O. Sepúlveda-Moreno, Manuel G. Ballesteros-Monrreal, Dora E. Valencia-Rivera, Gustavo A. González-Aguilar, Miguel A. Martínez-Téllez, Jesús Fernando Ayala-Zavala

**Affiliations:** 1 Coordinación de Tecnología de Alimentos de Origen Vegetal, Centro de Investigación en Alimentación y Desarrollo, A.C., Hermosillo 83304, Mexico; 2 Departamento de Ciencias Químico-Biológicas y Agropecuarias, Universidad de Sonora, Unidad Regional Norte, Campus Caborca, Caborca 83621, Mexico; 3 Departamento de Ciencias Económico-Administrativas, Universidad de Sonora, Unidad Regional Norte, Campus Caborca. H., Caborca 83621, Mexico

**Keywords:** antibiotics, food safety, pathogenic strains, multidrug-resistance bacteria

## Abstract

Pathogenic strains of *Escherichia coli* threaten public health due to their virulence factors and antibiotic resistance. Additionally, the virulence of this bacterium varies by region depending on environmental conditions, agricultural practices, and the use of antibiotics and disinfectants. However, there is limited research on the prevalence of antibiotic-resistant *E. coli* in agriculture. Therefore, this research aimed to determine the antibiotic resistance of *E. coli* isolated from the Honeydew melon production system in Hermosillo, Sonora, Mexico. Thirty-two *E. coli* strains were isolated from 445 samples obtained from irrigation water, harvested melons, the hands of packaging workers, boxes, and discarded melons. The resistance profile of the *E. coli* strains was carried out to 12 antibiotics used in antimicrobial therapeutics against this bacterium; a high level of resistance to ertapenem (100%) was detected, followed by meropenem (97%), and ampicillin (94%); 47% of the strains were classified as multidrug-resistant. It was possible to identify the prevalence of the extended-spectrum β-lactamase (ESBLs) gene *bla_TEM_* (15.6%), as well as the non-ESBL genes *qepA* (3.1%) and *aac(6′)lb-cr* (3.1%). The *E. coli* strains isolated from irrigation water were significantly associated with resistance to aztreonam, cefuroxime, amikacin, and sulfamethoxazole/trimethoprim. Irrigation water, packing workers’ hands, and discarded melons showed a higher prevalence of antibiotic-resistant, ESBL, and non-ESBL genes of *E. coli* strains in a farm and packing facility of Honeydew melon in Hermosillo, Sonora.

## 1. Introduction

Food safety has severe implications for the global economy and public health. *Escherichia coli* is among the top foodborne pathogens linked to several outbreaks yearly [1]. This bacterium is a frequent inhabitant of the gastrointestinal tract of animals, including humans; it is released into the feces and has therefore been used as an indicator of fecal contamination [2]. In addition, the plasticity of its genome has led to the evolution of this organism towards pathogenic strains capable of adapting to different niches and causing various diseases [3]. Vegetable contamination with *E. coli* can occur during crop cultivation, processing, distribution, marketing, or preparation [4]. Between 2018 and 2021, eight outbreaks were reported as being caused by the consumption of plant foods contaminated with pathogenic *E. coli* strains [1]. In addition to the infectious processes caused by *E. coli*, its treatment using conventional antibiotics is losing efficacy, making this challenge harder to solve.

*E. coli* has been identified in several food production systems, such as fruits, vegetables, poultry, cattle, pigs, and fish [5,6,7]. *E. coli* can pose an infectious risk to human health; furthermore, the problem is intensified if strains develop antibiotic resistance. Antibiotic-sensitive bacteria can acquire resistant determinants through a horizontal gene transfer of mobile elements (such as insertion sequences, transposons, plasmids, DNA bacteriophages, and pathogenicity islands) [8]. The transfer of antibiotic resistance genes from bacteria in the environment to those in the human tract represents a significant concern. It can occur, for example, through consuming food contaminated with drug-resistant species [9]. Therefore, knowing the antibiotic resistance profile of bacterial infections is challenging for prescribing the correct drugs.

Identifying antibiotic-resistant *E. coli* strains in food intended for human consumption is essential for public health and food safety. Much research has been conducted on antibiotic-resistant *E. coli* in animal-producing environments; however, few investigations are available on whether plant-based food-production chain environments can act as carriers or reservoirs for this microorganism. An outbreak associated with the consumption of fresh vegetables contaminated with enterohemorrhagic *E. coli* O104, capable of producing Shiga toxins and extended-spectrum β-lactamases was reported in Germany (2011) [10]. In a study conducted in South Korea in plant-based foods, the *mcr-1* gene was identified in 0.076% of the isolated *E. coli* strains; this finding is of great importance because this gene confers resistance to colistin, an antibiotic used as a last resort against multidrug-resistant *E. coli* in hospitalized patients [11]. Therefore, identifying the regional prevalence of antibiotic-resistant *E. coli* strains is essential to detecting the occurring horizontal gene transfer events and understanding the distribution of circulating resistance determinants threatening consumers’ health [12]. In addition, this information could be useful in designing specific actions to improve the correct use of antibiotics in the studied region. Consequently, this study aimed to determine the antibiotic resistance profiles and the distribution of *E. coli* strains isolated from irrigation water, soil, farmers’ hands, harvested melons, washing water, washed melons, packing tables’ surfaces, packers’ hands, cardboard packing boxes, and low-quality/damaged discarded melons in a local Honeydew melon farm in Hermosillo, Sonora, Mexico.

## 2. Results and Discussion

### 2.1. Identified Strains of E. coli

The samples (445) were obtained from a local farm and packing facility of Honeydew melon in Hermosillo, Sonora, Mexico. These samples were collected between May and June 2021 from irrigation water, soil, workers’ hands in the cropland, harvested melons, washing water, washed melons, packing tables’ surfaces, packing workers’ hands, cardboard packing boxes, and low-quality/damaged discarded melons. Thirty-two strains (7.2%) of *E. coli* were confirmed from the 445 samples obtained from a farm and packing facility of Honeydew melon in Hermosillo, Sonora. The prevalence rates of *E. coli* isolated from the irrigation water (59%), followed by low-quality discarded melons (29%), the hands of packaging workers (6%), harvested melons (3%), and cardboard packaging boxes (3%) are shown in Table 1.

The highest prevalence of *E. coli* strains was found in irrigation water (59%). The results are similar to the findings of Corzo et al. [5] in Northern Mexico, where they found *E. coli* in the water reservoir and irrigation lines in the production system of jalapeño pepper (18.8%, 7.7%), tomato (37.6%, 28.5%), and cantaloupe (7.1%, 21.4%), respectively. The analyzed farm of Honeydew melon has an open water reservoir, and this shallow water is the biggest challenge for fruit and vegetable production due to its exposure to contaminating vectors [13]. Deeper wells tend to be less contaminated than shallow waters due to restricted microbial infiltration [14]. The presence of *E. coli* in irrigation water might be due to several vectors, such as wild animals, manure, dust particles, tools, equipment, workers, and a depletion of the desired disinfectant concentration [14]. Another potential source contaminating irrigation water may be farm animals’ feces with multidrug resistance *E. coli*, due to the widespread use of antibiotics for treating *E. coli*-induced diseases in farm animals. In addition, *E. coli* has become one of the bacterial sources for multidrug-resistant genes, which have been prevalent and show an increasing trend [15].

The three main areas commonly contaminated in a packing facility are the reception area for the raw materials, processing areas (belts, rollers, and tables), and distribution areas [16]. Packers’ tools and the traffic of workers coming from the cropland to the packing facility also might influence cross-contamination [17]. The analyzed packing facility in this study had a large flow of workers and a lack of well-established hygiene and safety guidelines in the raw materials reception area.

Six percent of the *E. coli* strains were isolated from the packing house workers’ hands, and similar values were obtained in a study conducted on jalapeño (4.2%) and cantaloupe (5.7%) grown on farms in Northern Mexico [5]. Another sampling site with *E. coli* contamination was discarded melons (29%); this could be due to fecal contamination by wild animals or flies. Flies can carry pathogens on their legs, parts of their mouth, intestinal tract, and exoskeleton [18]. Pathogens detected in flies include diarrheagenic *E. coli*, *Cryptosporidium*, *Giardia lamblia*, *Norovirus*, *Salmonella*, *Shigella*, and *Vibrio cholerae* [19,20]. Discarded melons in this cropland are usually required for animal feeding; if no mechanical damage is noticed, this fruit is sometimes donated. Therefore, special care when disinfecting the products is recommended.

However, there were sampling sites from the farm and packing facilities from Honeydew melons where *E. coli* was not observed (soil, workers’ hands during harvest, washing water, washed melons, and packing tables’ surfaces), possibly due to several factors affecting bacterial growth. For example, no *E. coli* strains were isolated in the soil; this could be attributed to its moisture, texture, pH, and electrical conductivity, which influence the microbiota composition and pathogen survival [21]. In addition, it has been shown that the breakdown of *E. coli* O157:H7 may be influenced by land use factors, including soil pH, sand content, and organic matter [22]. The soil microbial community has also been shown to influence pathogenic bacteria’s survival, attributed to the progressive increase in competition for resources and antagonistic interactions associated with greater diversity. Similarly, enteropathogens’ physiological properties and life cycle influence their ability to survive within the soil matrix [23].

Another sampling site where *E. coli* was not observed was the water used for washing; this had a 200 ppm sodium hypochlorite concentration. Various research has shown that sodium hypochlorite uses different mechanisms against bacteria, including conformational changes in proteins and enzyme denaturation due to forming N-Cl bonds. It can also oxidize various enzymes, such as dehydrogenases or those involved in respiration [24]. In Honeydew melons, after the washing stage (sodium hypochlorite 200 ppm), *E. coli* was not observed, possibly due to the disinfectant solution used.

Good agricultural practices should be reinforced during the melon production chain to reduce the microbial contamination risk. Specifically, it has been recommended to bolster the disinfection procedures of workers’ hands, use hair nets or caps, and build mounds, wind chillers, and trenches to reduce runoff from animal production areas and waste management operations [25]. Another strategy offered to the analyzed farm was installing fences, removing vegetation and waste materials around the packing house, and implementing a pest control program.

### 2.2. Antibiotic Susceptibility of the Isolated E. coli Strains

**Figure 1:**
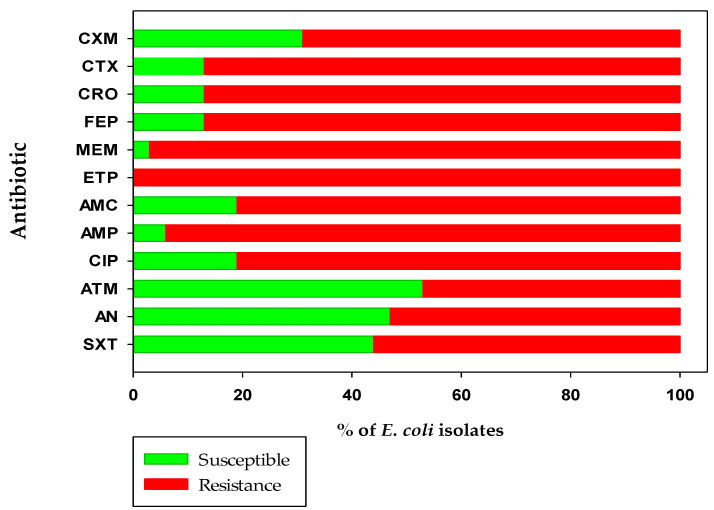
Susceptibility and resistance profile of *E. coli* strains isolated from the farm and packing facilities from Honeydew melon in Hermosillo, Sonora. Cefuroxime (CXM), cefotaxime (CTX), ceftriaxone (CRO), cefepime (FEP), meropenem (MEM), ertapenem (ETP), amoxicillin/clavulanic acid (AMC), ampicillin (AMP), ciprofloxacin (CIP), aztreonam (ATM), amikacin (AN), and sulfamethoxazole/trimethoprim (STX).

Antibiotic susceptibility tests showed that the isolated *E. coli* strains showed different resistance patterns to the 12 tested antibiotics, including four cephems (cefuroxime, cefotaxime, ceftriaxone, cefepime), two penems (meropenem, ertapenem), one penicillin with an inhibitor (amoxicillin/clavulanic acid), one penicillin (ampicillin), one quinolone (ciprofloxacin), one monobactam (aztreonam), one aminoglycoside (amikacin), and one sulphonamide (sulfamethoxazole/trimethoprim). The isolated strains were highly resistant to the carbapenems (ertapenem 100%, meropenem 97%), penicillin (ampicillin 94%), cephem (cefotaxime 87%, ceftriaxone 87%, cefepime 87%), and quinolones (ciprofloxacin 81%). For the three categories of antibiotics, *E. coli* strains were more susceptible to monobactam (53%), aminoglycoside (47%), and sulfonamide (44%) (Figure 1).

**Figure 2:**
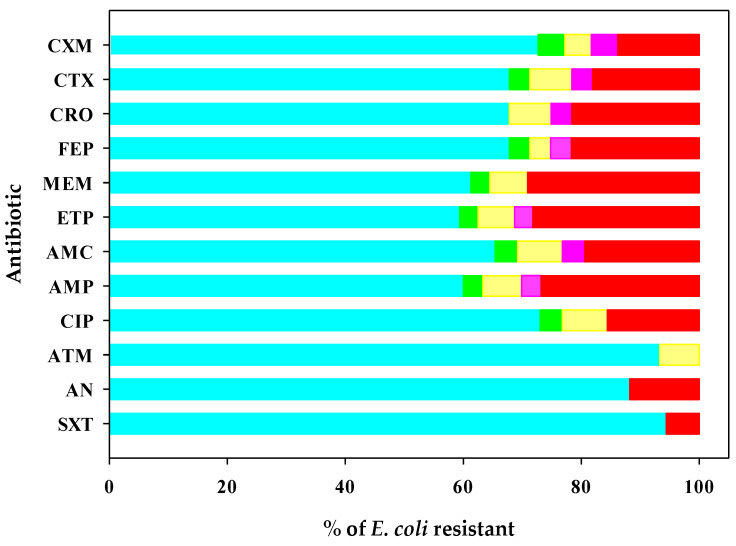
Antimicrobial resistance depends on sampling *E. coli* isolates from 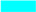 irrigation water, 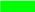 harvested melon, 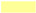 packing workers’ hands, 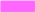 cardboard packing boxes, and 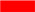 discarded melon from the Honeydew melon farm and packing facility in Hermosillo, Sonora. Cefuroxime (CXM), cefotaxime (CTX), ceftriaxone (CRO), cefepime (FEP), meropenem (MEM), ertapenem (ETP), amoxicillin/clavulanic acid (AMP), ampicillin (AMP), ciprofloxacin (CIP), aztreonam (ATM), amikacin (AN), and sulfamethoxazole/trimethoprim (STX).

**Figure 3:**
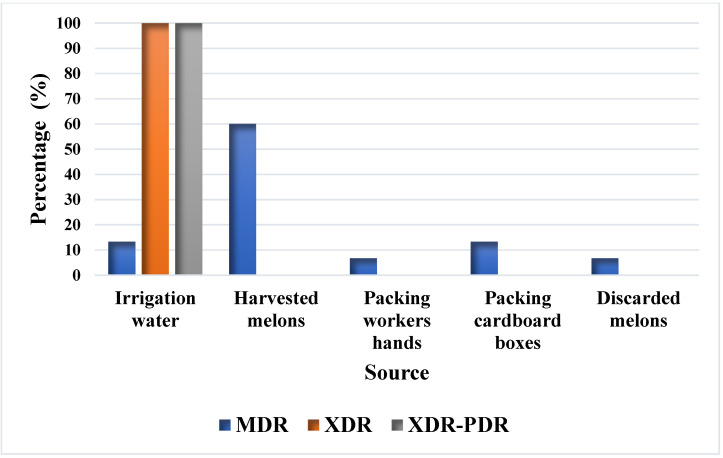
Percentages of *E. coli* strains with specific antibiotic resistance isolated from different points of the Honeydew melon farm and packing facility in Hermosillo, Sonora. MDR: multi-drug-resistant; XDR: extremely resistant; XDR-PDR: extremely resistant with a tendency to be pandrug-resistant.

This study showed that irrigation water had the highest prevalence of multidrug-resistant *E. coli* strains, followed by discarded melons, cardboard packaging boxes, harvested melons, and workers’ hands (Figure 2). The antibiotic-resistant profiles of *E. coli* isolated from discarded melons indicated the presence of multidrug-resistant strains (MDR), defined as being resistant to an antimicrobial agent in three or more categories. Meanwhile, extremely drug-resistant (XDR) strains that exhibited susceptibility to two types of antibiotics were detected mainly in the irrigation water. There were also *E. coli* strains resistant to the 12 antibiotics used in this research; these were found in the irrigation water and classified as extremely resistant strains with a tendency to be pandrug-resistant (XDR-PDR) (Figure 3). Antimicrobial resistance is a problem that limits treatment, and the severity of the disease may be due to the expression of virulence factors.

Table 2 shows the resistotype, ESBL, and non-ESBL of the *E. coli* strains isolated from the Honeydew melon farm and packing facility in Hermosillo, Sonora. Thirty-two *E. coli* strains were resistant to five or more categories of antibiotics, classifying them as multidrug-resistant. When determining ESBL genes, the *bla_TEM_* gene (15.6%) was the most prevalent, detected mainly in strains isolated from the irrigation water. The single genes associated with antibiotic resistance (*bla_CTX-M151_*, *bla_CTX-M1_*_&*8*_, and *bla_CTX-M9_*,) and several gene associations (*bla_CTX-M1_*_&*8*_ with *bla_TEM_*, *bla_CTX-M1_*_&*8*_ with *bla_CTX-M151_*, and *bla_CTX-M9_* with *bla_TEM_*) were identified in the *E. coli* isolated from irrigation water, discarded melon, and harvested melon. In the same way, it was possible to identify the presence of *E. coli* strains with quinolones (*aac (6′)-lb-cr* and *qepA*) resistance genes in the irrigation water and harvested melon. These results emphasize that strains of *E. coli* that carry a combination of antibiotic-resistant genes pose a clinical threat due to human infection.

*E. coli* uses different resistance mechanisms; among them is the acquisition of genes that encode for extended-spectrum β-lactamases (which confer resistance to broad-spectrum cephalosporins and other β-lactam drugs), 16S rRNA methylases (which confer pan-resistance to aminoglycosides), and *mcr* genes (which confer resistance to polymyxins) [26]. One of the most studied mechanisms of antibiotic resistance is the production of extended-spectrum β-lactamases, which can be divided into four groups: TEM, SHV, OXA, and CTX-M. These ESBL groups are responsible for resistance to broad-spectrum penicillins, cephalosporins, and monobactams. The *bla_TEM_*, *bla_SHV_*, *bla_OXA_*, and *bla_CTX-M_* gene families encoded ESBLs. These can spread between bacterial isolates by exchanging plasmids (and other mobile elements), which may harbor additional antimicrobial-resistant genes [27]. In this study, it was possible to identify the presence of no-ESBL(*qepA*, and *aac(6′)-lb-cr*) genes in the *E. coli* strains isolated from the irrigation water and the harvested Honeydew melons (Table 2). The *aac(6′)-lb-cr* gene provides resistance to ciprofloxacin; it is considered a variant of aac(6′)-lb (resistant to kanamycin, amikacin, and tobramycin) with two amino acid substitutions, which allows it to acetylate and reduce the activity of norfloxacin and ciprofloxacin [28]. This study detected *E. coli* strains without the targeted antibiotic resistance genes; however, they might harbor other resistance mechanisms.

### 2.3. Risk Analysis at Different E. coli Sampling Sites

Based on the evidence obtained in this research, it was possible to infer an association between the irrigation water and other isolation sites (harvested melons, packing workers’ hands, packing boxes, and discarded melons) with the frequency of the presence or absence of *E. coli* resistance to the following antibiotics: aztreonam, cefuroxime, amikacin, and sulfamethoxazole/trimethoprim. It is important to note that the rest of the antibiotics used in the research did not provide a significant association (Table 3).

Risk indicators and ODDS were used to establish the following associations: (i) It was 9.5, 1.8, 5.1, and 11.6 times more likely to identify aztreonam, cefuroxime, amikacin, and sulfamethoxazole/trimethoprim-resistant *E. coli* strains in irrigation water than in other sampling sites. (ii) There was a 65, 38, 63, and 81% higher risk of finding aztreonam, cefuroxime, amikacin, and sulfamethoxazole/trimethoprim-resistant *E. coli* strains in irrigation water than in other sampling sites, respectively. (iii) It was 2.8 times more likely to identify strains of *E. coli* resistant to aztreonam, cefuroxime, amikacin, and sulfamethoxazole/trimethoprim in irrigation water than non-resistant strains isolated from the same sampling site. (iv) It was 33, 6, 20, and 102 times more likely to find *E. coli* strains resistant to aztreonam, cefuroxime, amikacin, and sulfamethoxazole/ trimethoprim in irrigation water, respectively, compared to the total samples.

The choice of antibiotics and their usage pattern in agri-food production suggests a geographical variation influenced by production systems, the type and purpose of agriculture, legislative framework, and the socioeconomic status of the population and farmers [29,30]. Due to the indiscriminate use of antibiotics, they can be released into the environment through different sources, such as human waste streams and farming use [31]. It has been shown that administered antibiotics are not fully metabolized and are released unchanged into the environment, where their rate will depend on their specific type and administered dose [32]. This extended presence of antibiotics in the environment has promoted the development of resistant bacteria.

Susceptible bacteria have developed resistance to antibiotics by modifying their target binding sites, becoming neutralized through enzymes, or via changes in membrane permeability produced by the presence of efflux pumps [33,34]. In addition, bacteria can acquire antibiotic-resistant genes from other bacteria or phages through horizontal gene transmission [35]. This study demonstrated the prevalence of *E. coli* strains resistant to ciprofloxacin (81%, class quinolones), a broad-spectrum antimicrobial against many Gram-negative bacteria. As fluoroquinolone use has increased, more and more cases of quinolone-resistant strains have been reported. The resistance of *E. coli* strains to quinolones is a major medical threat because therapeutic options to treat infections caused by this bacterium are limited.

There are several studies on foods in which the presence of *E. coli* strains resistant to antibiotics is detected; for example, a study conducted by Mohamed et al. [36] showed resistance to kanamycin (77.8%), chloramphenicol (11.1%), streptomycin (100%), tetracycline (100%), and ciprofloxacin (5.6%) in strains isolated from lettuce, basil, beans, and cabbage. In Quito-Ecuador, hyperepidemic strains of *E. coli* ST410-A resistant to quinolones, which harbor the *bla_CTX-M15_* gene, were detected in alfalfa, lettuce, parsley, and coriander. The same study found the highest load of ESBL-producing *E. coli* in alfalfa [37]. When isolating *E. coli* strains from vegetables (lettuce, potato, carrot, tomato) in Tunisia, high rates of antibiotic resistance were observed: amoxicillin (68.7%), amoxicillin/clavulanic acid (73.7%), gentamicin (68.7%), kanamycin (66.2%), nalidixic acid (36.2%), streptomycin (68.7%), and tetracycline (35%) [38]. These studies showed that *E. coli* isolated from vegetables might be a reservoir of genes encoding antibiotic resistance. However, little attention is directed to studying the relationship between certain antibiotics used in specific food chains and the isolated strains’ resistance [39].

Taylor and Reeder [39] analyzed data from agricultural prescriptions; this study included regions from 32 countries and 436,674 records collected over eight years. They found that a proportion of 0.38% of the total records recommended at least one antibiotic in plant crops. The data recorded a total of 11 antibiotics used in crop plants, belonging to eight recommended classes, including kasugamycin (aminoglycoside), ningnanmycin (nucleoside), oxolinic acid (quinolone), validamycin (validamycin), and aureofungin (heptacene) which are mainly used exclusively in agricultural contexts. Meanwhile, others, such as amoxicillin (penicillin), tetracycline (tetracyclines), oxytetracycline (tetracyclines), gentamicin (aminoglycosides), streptomycin (aminoglycosides), and cefadroxil (cephalosporins), are also prescribed for animals and humans. Streptomycin was the most recommended antibiotic, followed by tetracycline and kasugamycin. Antibiotics were prescribed in more than 100 crop varieties, and rice received the highest number of recommendations, followed by tomato. Even when 60% of these prescriptions were directed to bacterial diagnosis, 12% were recommended against insects and mites, which highlights the relevance of improving the knowledge of specialists and farmers and avoiding the misuse of these drugs.

The biggest concern for antibiotic use in agriculture is the possibility of resistance spreading among bacterial species and eventually to human pathogenic strains. For this reason, the Food and Agriculture Organization of the United Nations, in its plan for antimicrobial resistance, recommends improving the awareness, capacity-building, monitoring, and use of antimicrobials in food and agriculture, as well as the promotion of good practices in food and agricultural systems and the prudent use of antimicrobials [40].

## 3. Conclusions

Irrigation water, packing workers’ hands, and discarded melon showed a higher prevalence of antibiotic-resistant, ESBL genes, and non-ESBL genes of *E. coli* strains in a Honeydew melon farm and packing facility in Hermosillo, Sonora. These strains showed resistance against ertapenem, meropenem, ampicillin, cefotaxime, and cefepime. In addition, a high prevalence of resistant multidrug *E. coli* strains was found. Therefore, these results suggest the need to reinforce the cautious use of antibiotics and good agricultural and processing practices to control the transmission of these strains.

## 4. Materials and Methods

### 4.1. Collection of Samples

Samples (445) were obtained from a local Honeydew melon farm and packing facility in Hermosillo, Sonora, Mexico. These samples were collected between May and June 2021 from irrigation water (11.2%), soil (11.2%), workers’ hands in the cropland (5.7%), harvested melons (11.2%), washing water (11.2%), washed melons (11.2%), packing tables’ surfaces (11.2%), packing workers’ hands (4.7%), packing cardboard boxes (11.2%), and low quality/damaged discarded melons (11.2%).

### 4.2. Isolation and Biochemical Characterization of E. coli

The samples were inoculated on MacConkey (Becton, Dickson and Company Sparks, MD, USA) and Eosin Methylene Blue (Becton, Dickson and Company, Sparks, MD, USA) agar plates, and both were incubated for 18 h at 37 ± 2 °C. Subsequently, biochemical identification was confirmed by tests of indole, mobility, sulfhydric acid production, glucose fermentation, lactose fermentation, gas production, Simmons citrate, methyl red, Voges-Proskauer, lysine decarboxylase, and urea [41].

### 4.3. Genomic DNA Extraction

The strains with biochemical characteristics of *E. coli* were subjected to DNA extraction by alkaline lysis according to the established protocols of The Molecular Cloning Laboratory Manual 2012 [42]. DNA extraction was preserved at −20 °C.

### 4.4. Molecular Identification of E. coli

The presumptive *E. coli* strains were identified with conventional PCR using GoTaq Green Master Mix (Promega, WI, USA), searching for the allantoin gene (*ybbW*) specific for this species; the used oligonucleotides in this study are listed in Table S1 [43,44]. The PCR product was observed by electrophoresis on a 1% agarose gel in 1× TAE buffer stained with GelStarTM Stain (Lonza, Morristown, NJ, USA). Once identified, the *E. coli* strains were stored in Luria-Bertani broth (Becton, Dickson and Company, Sparks, MD, USA) with glycerol (20% *v/v*) and kept at −80 °C for the antibiotic susceptibility test [45].

### 4.5. Antibiotic Resistance

The antibiogram was performed using the Kirby–Bauer disc diffusion method [46], in which the tested substances were the most common antibiotics prescribed against *E. coli* infections, including cefotaxime (31 μg), aztreonam (30 μg), cefuroxime (30 μg), ceftriaxone (32 μg), ampicillin (10 μg), cefepime (30 μg), amoxicillin/clavulanic acid (20/10 μg), amikacin (30 μg), ciprofloxacin (5 μg), meropenem (10 μg), sulfamethoxazole/trimethoprim (1.25/23.75 μg), and ertapenem (10 μg). For the test, the isolated PCR-identified *E. coli* strains and *E. coli* ATCC 25922 (control) were cultured in Mueller Hinton broth and incubated at 35 °C ± 2 °C for 16–18 h. A bacterial suspension was prepared at 1.5 × 10^8^ CFU/mL and was plated on Mueller–Hinton agar plates. After 15 min of drying, antibiotics discs were placed, followed by incubation at 35 °C ± 2 °C for 16–18 h. The inhibition zones were analyzed following the guidelines of the Clinical and Laboratory Standards Institute (2021) to establish if the strain was resistant or susceptible to antibiotics [46]. Additionally, Magiorakos’ criteria were followed in classifying the *E. coli* strains as non-multidrug resistant (NMDR), multidrug-resistant (MDR), extremely resistant (XDR), or pandrug resistant (PDR) [47].

### 4.6. Molecular Identification of Extended Spectrum β-Lactamases (ESBLs) and Non-ESBL Genes

The presence of antibiotic resistance genes was searched for in the identified *E. coli* strains using conventional PCR with GoTaq Green Master Mix (Promega) and Table S1 shows the specific primers for the identification of ESBL genes (*bla_CTX-M1_*_&*8*_, *bla_CTX-M2_*, *bla_CTX-M9_*, *bla_CTX-M151_*, *bla_TEM_*, *bla_SHV_*) and non-ESBL genes (*qepA* and *aac(6′)-lb-cr*). Each PCR reaction was performed using 12.5 µL GoTaq Green Master Mix, 0.5 µL of each primer (10 µM), 1.5 µL [50–75 ng] of template DNA, and necessary distilled water to obtain a final volume of 25 µL. The PCR product was observed by electrophoresis on a 1% agarose gel electrophoresis.

### 4.7. Disposal of Microorganisms and Reagents

Disposal of microorganisms and reagents was carried out following the Official Mexican Standard NOM-087-SEMARNAT-SSA1-2002 and NOM-052-SEMARNAT-2005.

### 4.8. Statistical Analysis

Descriptive statistics were used to determine the prevalence of *E. coli* strains and phenotypic antibiotic resistance at different points of the Honeydew melon production system. Subsequently, Pearson’s chi-square test with explanatory scope based on risk indicators and ratios was obtained and included relative risk (RR), attributable risk (AR), odds exposed (ODD-E), odds unexposed (ODD-NE), and odds ratio (ODD-R). Data were analyzed with IBM SPSS Statistics (Version 21.0).

### 4.9. Ethical Considerations

Ethical approval was obtained from the Comité de Ética en Investigación del CIAD (CEI/011/2021) regarding the procedure to isolate bacteria from the local farm and packing house. Participants were informed of the study’s aim, and interested volunteers signed the informed consent form.

## Figures and Tables

**Table 1:** Presumptive and confirmed *E. coli* strains isolated from the Honeydew melon farm and packing facility.

		*E. coli* Strains	
Source	No. of Samples	Presumptive	Confirmed
Irrigation water	50	44 (51%)	19 (59%)
Soil	50	6 (7%)	0 (0%)
Workers’ hands during harvest	25	3 (4%)	0 (0%)
Harvested melons	50	4 (5%)	1(3%)
Washing water	50	0 (0%)	0 (0%)
Washed melons	50	1 (1%)	0 (0%)
Packing table surfaces	50	4 (5%)	0 (0%)
Packing workers’ hands	20	3 (4%)	2 (6%)
Cardboard packing boxes	50	1 (1%)	1 (3%)
Discarded melons	50	20 (23%)	9 (29%)
Total	445	86 (19.3%)	32 (7.2%)

**Table 2:** Resistotype, ESBL genes, and non-ESBL genes among *E. coli* isolates obtained from the Honeydew melon farm and packing facility.

Isolate	Source	Resistotype	ESBL Genes	ESBL Non-Genes
Ec -A4-2	irrigation water	CTX, ATM, CXM, CRO, AMP, FEP, AMC, AN, CIP, MEM, SXT, ETP	---	---
Ec-A21-2	irrigation water	CTX, ATM, CRO, AMP, FEP, AMC, AN, CIP, MEM, SXT, ETP	*bla_CTX-M9_*, *bla_TEM_*	---
Ec-A32	irrigation water	CTX, ATM, CXM, CRO, AMP, FEP, AMC, CIP, MEM, SXT, ETP	*bla_TEM_*	---
Ec-A34	irrigation water	CTX, CXM, CRO, AMP, FEP, AMC, CIP, MEM, SXT, ETP	*bla_CTX-M151_*	---
Ec-A35-2	irrigation water	CTX, ATM, CXM, CRO, AMP, FEP, AMC, AN, CIP, MEM, SXT, ETP	*bla_TEM_*	---
Ec-A36	irrigation water	CTX, ATM, CXM, CRO, AMP, FEP, AMC, AN, CIP, MEM, SXT, ETP	---	---
Ec-A37	irrigation water	CTX, ATM, CXM, CRO, AMP, FEP, AMC, AN, CIP, MEM, SXT, ETP	---	---
Ec-A38	irrigation water	CTX, ATM, CXM, CRO, AMP, FEP, AMC, AN, CIP, MEM, SXT, ETP	---	---
Ec-A39	irrigation water	CTX, ATM, CXM, CRO, AMP, FEP, AMC, AN, CIP, MEM, ETP	*bla_CTX-M1-8_*, *bla_CTX-M151_*	---
Ec-A40	irrigation water	CTX, ATM, CXM, CRO, AMP, FEP, AN, CIP, MEM, SXT, ETP	---	---
Ec-A41	irrigation water	CTX, ATM, CXM, CRO, AMP, FEP, AMC, AN, CIP, MEM, SXT, ETP	---	---
Ec-A42	irrigation water	CTX, ATM, CXM, CRO, AMP, FEP, AN, CIP, MEM, SXT, ETP	*bla_CTX-M1-8_*	---
Ec-A44	irrigation water	CTX, ATM, CXM, CRO, AMP, FEP, AMC, AN, CIP, MEM, SXT, ETP	---	*aac (6′)-lb-cr*
Ec-A45	irrigation water	CTX, ATM, CXM, CRO, AMP, FEP, AMC, AN, CIP, MEM, SXT, ETP	---	---
Ec-A46	irrigation water	CTX, ATM, CXM, CRO, AMP, FEP, AMC, AN, CIP, MEM, SXT, ETP	---	---
Ec-A47	irrigation water	CTX, CXM, CRO, AMP, FEP, AMC, CIP, MEM, SXT, ETP	*bla_TEM_*	---
Ec-A48	irrigation water	CTX, CXM, CRO, AMP, FEP, AMC, CIP, MEM, SXT, ETP	---	---
Ec-A49	irrigation water	CTX, CRO, FEP, AMC, AN, CIP, MEM, ETP	---	---
Ec-A51	irrigation water	CTX, CRO, AMP, FEP, AMC, AN, CIP, MEM, SXT, ETP	*bla_CTX-M9_*	---
Ec-MR11	discarded melon	CTX, CXM, CRO, AMP, FEP, AMC, AN, CIP, MEM, ETP	---	---
Ec-MR15	discarded melon	CXM, AMP, FEP, AMC, MEM, SXT, ETP	---	---
Ec-MR16	discarded melon	CRO, FEP, CIP, MEM, ETP	---	---
Ec-MR17	discarded melon	CRO, AMP, AMC, AN, MEM, ETP	*bla_CTX-M1-8_*, *bla_TEM_*	---
Ec-MR23	discarded melon	CTX, AMP, FEP, MEM, ETP	---	---
Ec-MR25	discarded melon	AMP, MEM, ETP	---	---
Ec-MR28	discarded melon	CTX, CRO, AMP, FEP, AMC, CIP, MEM, ETP	*bla_TEM_*	---
Ec-MR34	discarded melon	CTX, CRO, AMP, FEP, CIP, MEM, ETP	---	---
Ec-MR35	discarded melon	CTX, CXM, CRO, AMP, AMC, MEM, ETP	---	---
Ec-MC46	harvested melon	CTX, CXM, AMP, FEP, AMC, CIP, MEM, ETP	*bla_TEM_*	*qepA*
Ec-MAE44	packing workers hands	CTX, CXM, CRO, AMP, FEP, AMC, CIP, MEM, ETP	---	---
Ec-MAE45	packing workers hands	CTX, ATM, CRO, AMP, AMC, CIP, MEM, ETP	---	---
Ec-C49	melon packing boxes	CTX, CXM, CRO, AMP, FEP, AMC, ETP	---	---

Cefotaxime (CTX), aztreonam (ATM), cefuroxime (CXM), ceftriaxone (CRO), ampicillin (AMP), cefepime (FEP), amoxicillin/clavulanic acid (AMC), amikacin (AN), ciprofloxacin (CIP), meropenem (MEM), sulfamethoxazole/trimethoprim (STX), ertapenem (ETP).

**Table 3:** *E. coli* antibiotic resistance inferences based on the sampling site.

Antibiotic	Sample	% Antibiotic Resistance of *E. coli*		Results						
		Yes	No	RR	AR	ODD-E	ODD-NE	ODD-R	*X* ^2^	*p*-Value
ATM	Irrigation water	73.7	26.3	9.5	0.65	2.8	0.08	33.6	13.4	0.001
	Others *	7.7	92.3							
CXM	Irrigation water	84.2	15.8	1.8	0.38	5.3	0.85	6.2	5.2	0.023
	Others	46.1	53.9							
AN	Irrigation water	78.9	21.1	5.1	0.63	3.7	0.18	20.6	12.5	0.001
	Others	15.4	84.6							
SXT	Irrigation water	89.5	10.5	11.6	0.81	8.5	0.08	102	20.9	0.001
	Others	7.7	92.3							

Relative risk (RR), attributable risk (AR), odds exposed (ODD-E), odds unexposed (ODD-NE), odds ratio (ODD-R), aztreonam (ATM), cefuroxime (CXM), amikacin (AN), sulfamethoxazole/trimethoprim (SXT). * Others: harvested melons, packing workers’ hands, packing boxes, and discarded melons.

## Supplementary Materials

The following supporting information can be downloaded at: https://www.mdpi.com/article/10.3390/antibiotics11121789/s1, Table S1: Specific oligonucleotides used in this study [48,49].
