# Supplementary material for: Prevalence of Antibiotic-Resistant E. coli Strains in a Local Farm and Packing Facilities of Honeydew Melon in Hermosillo, Sonora, Mexico
Source: Antibiotics (Basel). 2022 Dec 9;11(12):1789. doi: 10.3390/antibiotics11121789 (PMC9774811; doi:10.3390/antibiotics11121789)
Supplement: Supplementary file 1 [file antibiotics-11-01789-s001.zip › antibiotics-2055853-supplementary.pdf]

Table S1. Specific oligonucleotides used in this study

Gene	Sequence (5'- 3')	Amplicon size	Reference
ybbw	F:tgattggcaaaatctggccg<br>R:atactggcaatcagtacgcc	670 pb	[43,44]
CTX-M1&8	F:tgtgcagyaccagtaargykatg<br>R:tarrtsaccagaayvagcggc	583 pb	[48]
CTX-M2	F:cgagtggcagtaccagtaagg<br>R:cgatatcgttggtggtgc	540 bp	[48]
CTX-M9	F:atggtgacaaagagagtgcaa<br>R:aatatcattggtggtgccgtag	747 pb	[48]
CTX-M151	F:gcggccatgataggtacg<br>R:aaagtaagtcacaataaccagcg	786 pb	[48]
TEM	F:caacattttcgtgtcgccc<br>R:gcttaatcagtgaggcacc	844 pb	[48]
SHV	F:tattatctccctgttagcca<br>R:cgctctgctttgttattc	783 pb	[48]
qepA	F:gcaggtccagcagcggggtag<br>R: caactgcttgagcccgtag	617 pb	[49]
aac(6´)-lb-cr	F: ttgcgatgctctatgagtggcta<br>R:ctcgaatgcctggcgtgttt	482 pb	[49]
